# Lack of exon 10 in the murine tau gene results in mild sensorimotor defects with aging

**DOI:** 10.1186/1471-2202-14-148

**Published:** 2013-11-22

**Authors:** Astrid Gumucio, Lars Lannfelt, Lars NG Nilsson

**Affiliations:** 1 Department of Public Health and Caring Sciences, Rudbeck Laboratory, Uppsala University, SE-75185 Uppsala, Sweden; 2 Department of Pharmacology, Oslo University and Oslo University Hospital, Postboks 1057 Blindern, NO-0316 Oslo, Norway

**Keywords:** Alzheimer’s disease, Tau, Knockout mice, Alternative splicing, Microtubule

## Abstract

**Background:**

Complex species-specific, developmental- and tissue-dependent mechanisms regulate alternative splicing of *tau,* thereby diversifying tau protein synthesis. The functional role of alternative splicing of *tau* e.g. exon 10 has never been examined *in vivo,* although genetic studies suggest that it is important to neurodegenerative disease.

**Results:**

Gene-targeting was used to delete exon 10 in murine *tau* on both alleles (E10−/−) to study its functional role. Moreover, mice devoid of exon 10 (E10+/−) on one allele were generated to investigate the effects of 1:1 balanced expression of *4R-/3R*-*tau* protein, since equal amounts of 4R-/3R-tau protein are synthesized in human brain. Middle-aged E10−/− mice displayed sensorimotor disturbances in the rotarod when compared to age-matched E10+/− and wild-type mice, and their muscular grip strength was less than that of E10+/− mice. The performance of E10+/− mice and wild-type mice (E10+/+) was similar in sensorimotor tests. Cognitive abilities or anxiety-like behaviours did not depend on exon 10 in *tau*, and neither pathological inclusions nor gene-dependent morphological abnormalities were found.

**Conclusion:**

Ablation of exon 10 in the murine *tau* gene alters alternative splicing and tau protein synthesis which results in mild sensorimotor phenotypes with aging. Presumably related microtubule-stabilizing genes rescue other functions.

## Background

Tau belongs to the family of microtubule-associated proteins (MAPs), which bind to and/or interact with microtubule. It has been suggested that tau and other MAPs serve to promote assembly of microtubule to make them structurally stably, yet dynamic [1]. Disruption of the *tau* gene led to structural abnormalities of microtubule organization in small-calibre axons [2], and behavioural deficits with hyperactivity, impaired motor strength and coordination [3]. In contrast with the initial report [2], there were *in vitro* phenotypes with delayed development of neuronal polarity and formation of axons in cultured embryonal hippocampal neurons from tau knockout mice [4]. Functional redundancy, particularly between *tau* and *map* genes likely exists, since map1a protein expression was enhanced in tau knockout mice [2,4]. Indeed tau/map1b double knockout mice displayed brain anomalies with severe defects on axon tract and neuronal layer formation, together with abnormal growth cone morphology and pronounced microtubule disorganization in primary neurons derived from these mice [5]. Tau protein synthesis and function is regulated by alternative splicing in a complex species-specific, developmental- and tissue-dependent manner [6,7]. Depending upon the inclusion or exclusion of exon 10, *tau* mRNA isoforms with three (*3R-tau* mRNA) and four (*4R-tau* mRNA) microtubule binding domains are generated, and in total six *tau* mRNA isoforms are produced in the human brain. In the adult human brain, splicing is balanced with a 1:1 expression of *4R*- and *3R-tau* mRNA [8,9]. This is in contrast to mouse brain in which only *3R-tau* mRNA is generated at birth and only *4R-tau* mRNA is synthesized at adulthood [7,10,11]. Thus equal amounts of 4R- and 3R-tau protein is synthesized in adult human brain, while only 4R-tau protein is produced in adult mouse brain. Interestingly, mutations in exon 10 or in adjacent regulatory sequences, can give rise to neurodegenerative disease with accumulation of filamentous inclusions of tau in the human brain. Regulation of alternative splicing of *tau* is very complex and partly unknown. In order to investigate the functional role of exon 10 in murine *tau* we ablated the gene on both alleles, and thereby generated E10−/− mice which should in theory only synthesize 3R-tau protein. Moreover, the effect of a 1:1 balanced *3R-/4R-tau* mRNA splicing of human brain was examined by generating mice in which exon 10 in *tau* was deleted on only one allele (E10+/−). E10+/− mice should in theory synthesize equal amounts of 3R- and 4R-tau protein, as in the human brain (Additional file 1: Figure S1). The effects of fine-tuned regulation of alternative splicing being critical to maintain neuronal functions and viability could thereby be assessed [12]. Here we show that E10−/− but not E10+/− mice, display impaired sensorimotor abilities with aging.

## Results

### Generation of mice devoid of exon 10 in murine tau

**Figure 1:**
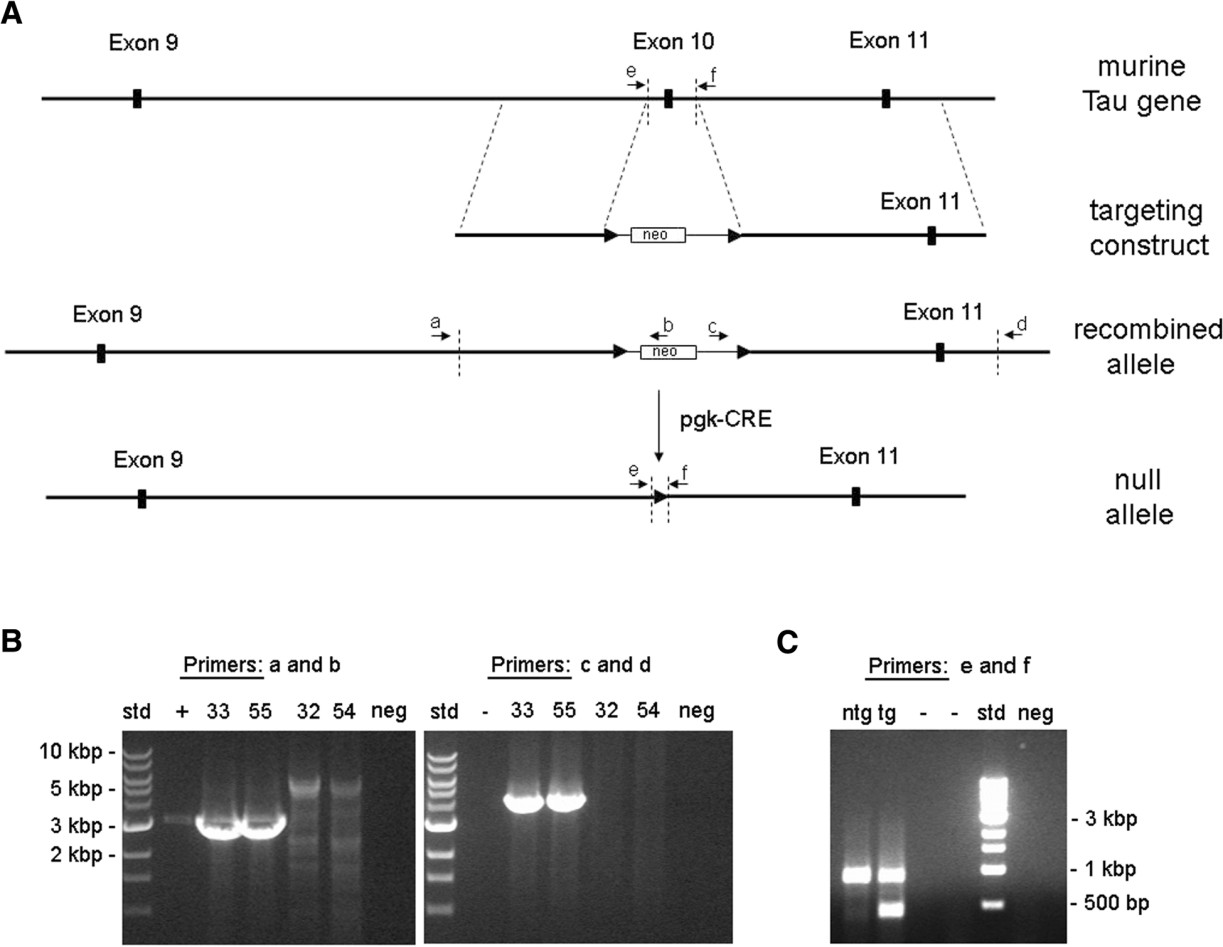
**Gene targeting strategy and verification. (A)** An outline of the murine tau gene (around exon 9–11) is shown, as well as the targeting construct and the modified murine tau gene which was generated by homologous recombination. The null allele, lacking exon 10, was generated by breeding mice harbouring the modified murine tau gene with pgk-CRE transgenic mice. **(B)** Identification of the recombined allele in genomic DNA of embryonic stem cell (ES) clones 33 and 55 by PCR-analyses. Bands of expected sizes, 3.0 kbp (with primers a and b) and 4.0 kbp (with primers c and d) were detected in these clones, but not in ES clones 32 and 54. **(C)** PCR analysis with primers flanking the deleted chromosomal region (primers e and f). Amplification of genomic DNA from E10+/− mouse (E10) resulted in two distinct bands, a 900 bp band from the endogenous *tau* gene and a 460 bp band from the null allele. There was only a 900 bp band from the endogenous *tau* gene in wild-type mouse (wt). Positive (+) and negative (neg) controls for the PCR reactions. Empty lanes (−).

Genomic regions (2.2 and 3.6 kbp) framing exon 10 in the murine *tau* gene were amplified with high-fidelity long-range PCR and subcloned into a pBluescript II KS-vector that harboured a loxP-neomycin-loxP-cassette (Figure 1A). The gene targeting construct was checked by multiple restriction enzyme digests and sequencing of both introns and the intervening neomycin cassette. The gene-targeting vector was made linear with SspI and introduced into R1 embryonic stem (ES) cell with electroporation. ES cell clones were screened for homologous recombination and in a few clones, integration in the murine *tau* locus led to deletion of exon 10 and adjacent intron sequences (~200 bp on each side of exon 10). The clones were screened with PCR-reactions in which one primer was located outside the homologous sequences of the targeting construct. Those experiments resulted in 3.0 kbp and 4.0 kbp bands respectively (Figure 1B). One of the positive ES cell clones was microinjected into blastocysts and transplanted into pseudopregnant mice. Male chimeric mice were bred with C57Bl/6 J and their offspring were examined for evidence of germ line transmission. These were further bred with transgenic mice expressing phosphoglycerate kinase (pgk)-CRE recombinase to delete the neo cassette. PCR with genomic DNA and primers flanking exon 10 provided evidence of an endogenous tau allele (900 bp) and a null allele (460 bp) in E10+/− mice, while only a single band was present in wild-type siblings (E10+/+; Figure 1C).

### Lack of exon 10 in tau results in 3R-tau protein synthesis in adult mouse brain

**Figure 2:**
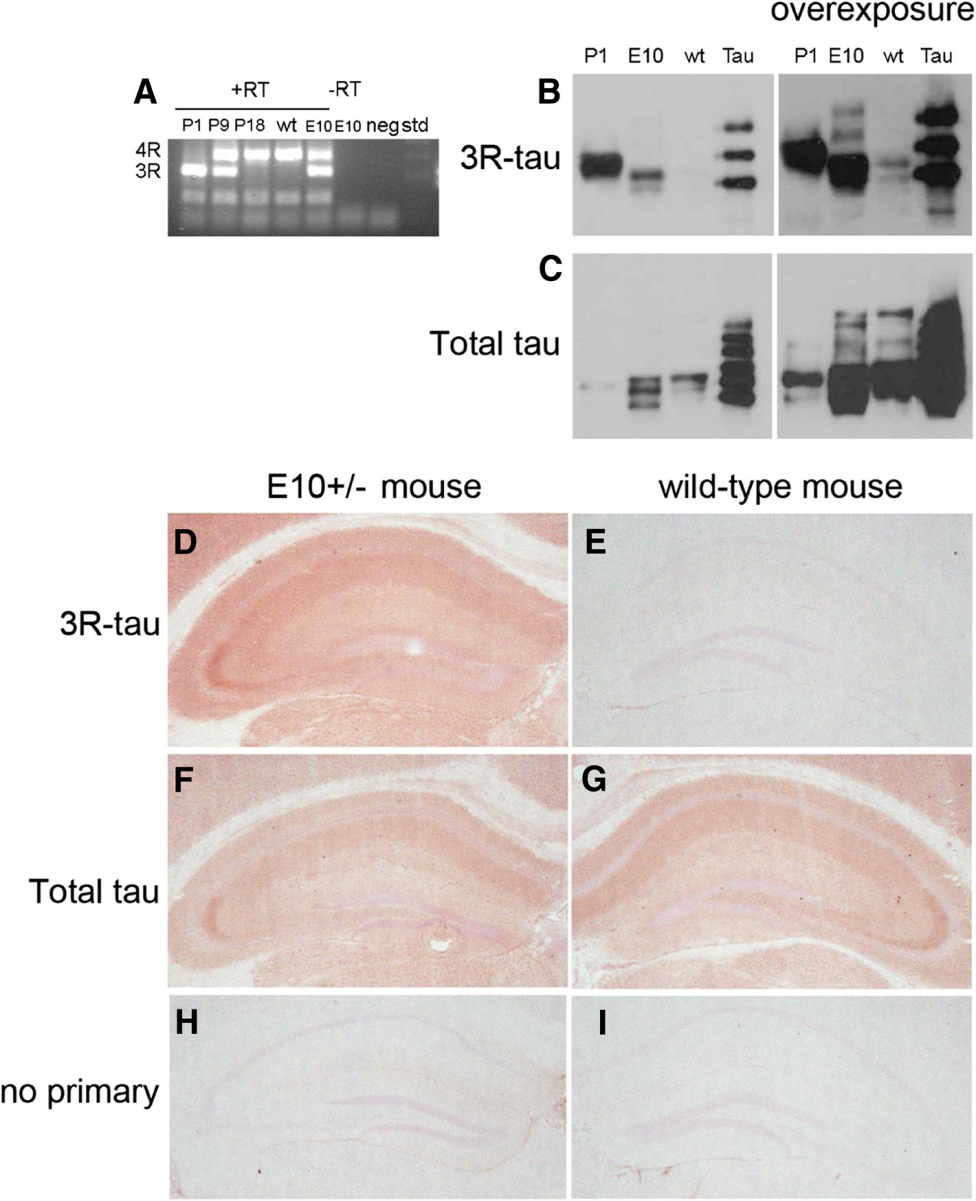
**Tau mRNA and protein expression in E10+/− and wild-type mice. (A)** Only *3R-tau* mRNA (212 bp) was expressed at birth (P1) in wild-type mice (wt). At (P9), *3R-tau* and *4R-tau* mRNA (305 bp) was expressed, while at (P18) and in 2-month-old wt-mice, exon 10 was included and *4R tau* mRNA was expressed. In contrast, 2-month-old E10+/− mice (E10) produced *3R-tau* and *4R-tau* mRNA in equal proportions. Signals depended on mRNA and reverse transcriptase in the cDNA synthesis reaction (−RT, E10). **(B)** Left; Western blot with a selective antibody for 3R tau resulted in a 38 kDa band in 2 months-old E10+/− mouse brain (E10). Right; Two additional bands were seen when films were overexposed. These bands likely represented tau-isoforms with N-terminal domains exon 2 and 3. In an age-matched wt-mouse there was only a small amount of 3R-tau protein. **(C)** Left; An antibody (Tau-1) that recognizes 3R-tau as well as 4R-tau generated two protein bands at 38 and 39 kDa in brains of 2-month-old E10+/− mice (E10). Right; Overexposure results in two additional double bands likely representing isoforms of tau with the N-terminal domains exon 2 and 3 of tau protein. Only a small amount of 3R tau protein was found in age-matched wild-type littermates (wt). Recombinant human tau protein representing all tau isoforms and a newborn wild-type mouse brain (P1) was included as a positive control. **(D)** Immunostaining with selective antibody 3RD showed that 3R-tau was mainly localized in axons of E10+/− mice, **(E)** but not present in an age-matched wild-type mice. **(F-G)** The non-selective antibody Tau-1, which recognizes both 3R-tau and 4R-tau, stained hippocampal layers which are rich in axons in both E10+/− **(F)** and wild-type mice **(G). (H-I)** No immunostaining in E10+/− **(H)** or wild-type mice in the absence of primary antibody **(I)**.

Next we investigated E10+/− mice for expected deviations in *tau* mRNA expression and protein synthesis. RT-PCR analyses were performed with primers located within exon 9 and exon 11 to generate PCR products of 305 bp (*4R-tau* mRNA) and 212 bp (*3R-tau* mRNA) respectively. As expected, a 1-day-old wild-type mouse (wt; P1) expressed only *3R-tau* mRNA, while a 9-days-old wild-type mouse (P9) generated equal amounts of *3R-* and *4R-tau* mRNA due to alternative splicing (Figure 2A). An 18-days-old wild-type mouse (P18) produced only *4R-tau* mRNA. In contrast, a 2-months-old E10+/− mouse (E10) generated *3R-* and *4R-tau* mRNA in essentially equal proportions, while only *4R-tau* mRNA was produced in an age-matched wild-type mouse (Figure 2A). Protein synthesis was examined with RD3 antibody, which is selective for 3R-tau, by western blot and immunohistochemistry. On western blot, all three isoforms of 3R-tau were found in E10+/− (E10) mouse brain, but the isoform devoid of exon 2 and 3 was the predominant (Figure 2B and Additional file 1: Figure S1). Tau-1, an antibody which recognizes both 3R- and 4R-tau, generated extra protein bands in E10+/− (E10) mice as compared to wild-type (wt) mice (Figure 2C). The results were consistent with 3R-tau protein synthesis in E10+/− (E10) mouse brain. Immunohistochemistry with RD3 antibody resulted in strong staining in the stratum radiatum in CA1-CA3 areas of the hippocampus and faint staining in the soma of pyramidal neurons in adult E10+/− mice (Figure 2D), but not in wild-type mice who did not express 3R-tau protein (Figure 2E). In contrast, the non-selective Tau-1 antibody showed a staining pattern consistent with tau being mainly confined to the axonal compartment [13] in both adult E10+/− (Figure 2F) and in wild-type mice (Figure 2G). There was no immunostaining in the absence of a primary antibody (Figure 2H-I).

### Absence of exon10 in tau results in age-dependent sensorimotor dysfunctions

**Figure 3:**
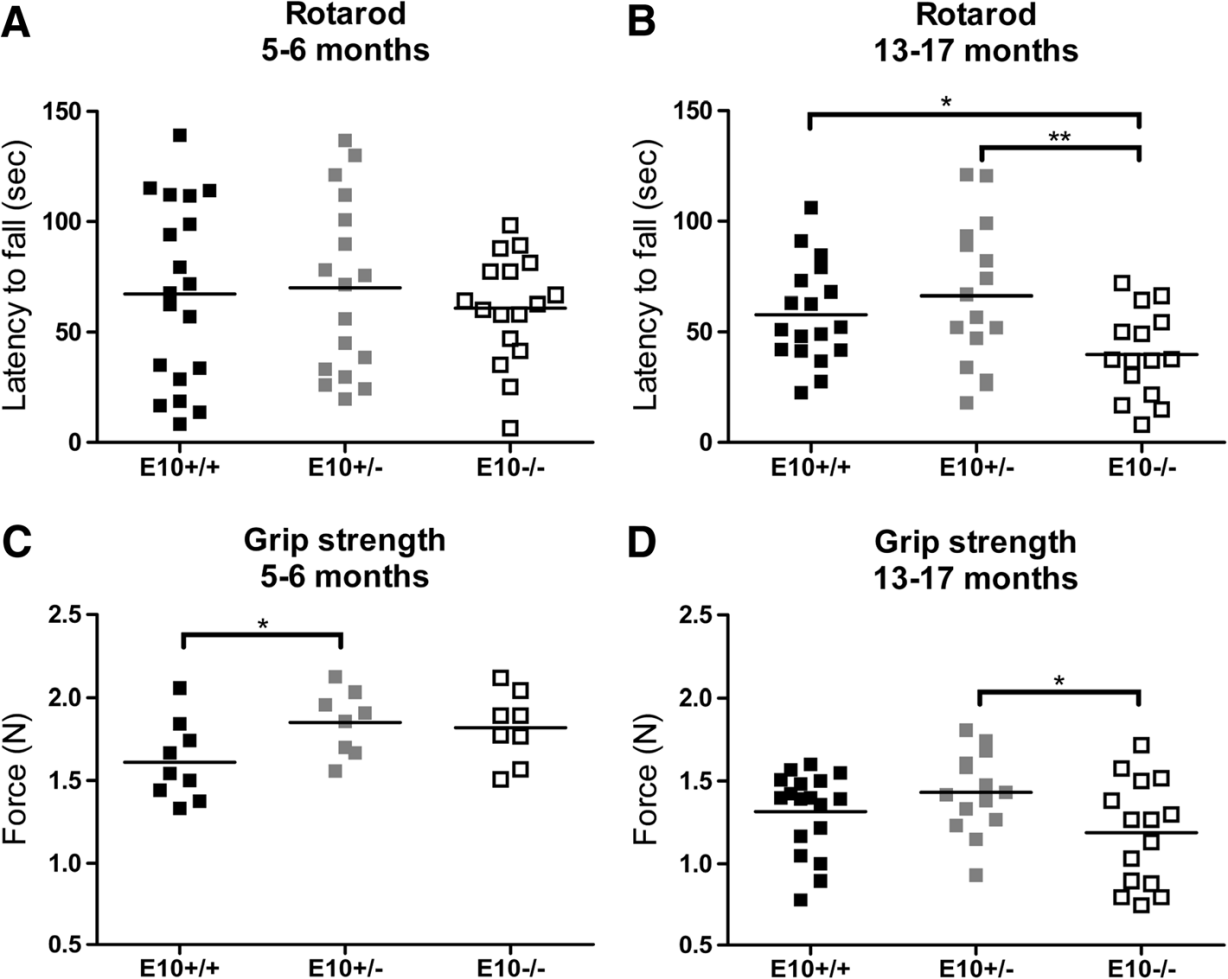
**Sensorimotor functions of wild-type, E10+/− and E10−/− mice. (A)** Mice devoid of tau exon 10 (E10−/−) showed no deficits in rotarod when they were 5–6 months-old (E10+/+, n = 19; E10+/−, n = 17; E10−/−; n = 17), **(B)** but when compared to wild-type (E10+/+, p < 0.05) and E10+/− (p < 0.01) their performance was impaired when they were 13–17 months-old (E10+/+, n = 18; E10+/−, n = 16; E10−/−; n = 15). **(C)** 5–6 months-old wild-type mice (E10+/+) were weaker than E10+/− (p < 0.05: E10+/+, n = 9; E10+/−, n = 8; E10−/−; n = 8), **(D)** while 13–17 months-old E10−/− mice were weaker than E10+/− (p < 0.05). *p < 0.05; **p < 0.01.

Subsequently, we aimed to investigate the physiological and functional role of exon 10 in *tau*. In previous reports, tau knockout mice developed sensorimotor dysfunctions due to loss of tau protein synthesis [3]. Cerebellum, which is implicated in balance and motor learning, develops at postnatal stages when 4R-tau is normally produced. We therefore examined whether partial or complete absence of exon 10 in *tau* led to sensorimotor anomalies in adult (5–6 months) and middle-aged (13–17 months) gene-manipulated mice. Rotarod and grip strength meter were used to assess sensorimotor dysfunctions. Genotype-dependent motor coordination dysfunction was not detected among adult mice (Figure 3A, F(2,50) = 0.27; p = 0.76), while differences in rotarod performance were observed among middle-aged mice (F(2,46) = 4.34; p < 0.05). Post-hoc analyses showed that E10−/− mice were impaired compared to E10+/+ (p < 0.05) and E10+/− (p < 0.01, Figure 3B). Similar results were found when the highest speed at which the animals remained on the rotarod was used as outcome measure (Additional file 1: Figure S2, F(2.46) = 5.05; p < 0.05). Post-hoc analyses revealed that the muscular strength of adult E10+/− mice was superior to that of E10+/+ mice (Figure 3C; p < 0.05). In contrast, middle-aged E10−/− mice were weaker than E10+/− (p < 0.05), but not E10+/+ mice (p = 0.10; F(2,45) = 2.43; Figure 3D).

### E10−/− mice are less active than E10+/− mice in IntelliCage

**Figure 4:**
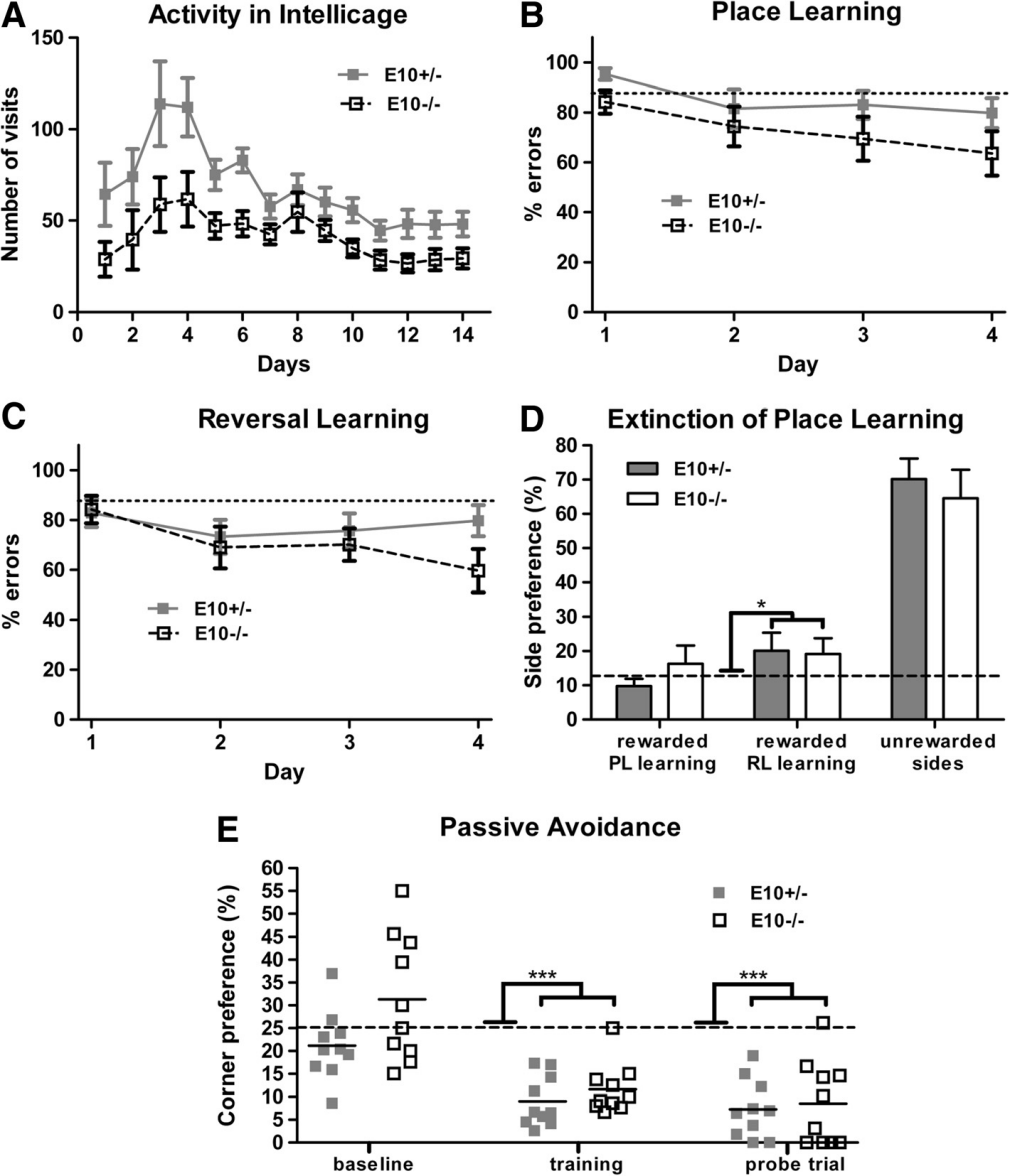
**Activity and learning ability of E10+/− and E10−/− mice in IntelliCage. (A)** 9–10 months-old mice devoid of tau exon 10 (E10−/−) were less active when habituating to IntelliCages as compared to E10+/− mice (E10+/−, n = 10; E10−/−; n = 12). No genotype-dependent differences were observed during **(B)** Place learning, **(C)** Reversal learning and **(D)** Extinction of place learning (E10+/−, n = 10; E10−/−; n = 10). **(D)** The results were indicative of response perseverance since the previously rewarded corner (rewarded RL learning; p < 0.001) was preferred when compared to chance level (dotted line, 12.5%) when all locations were again made accessible for drinking. **(E)** In passive avoidance, the mice significantly avoided those corners in which an aversive stimulus had been given during training (p < 0.001) and probe trial (p < 0.001) when compared to chance level (dotted line, 25%), i.e. the animals learnt and remembered but there were no genotype-dependent differences in associative learning or memory. *p < 0.05; **p < 0.01; ***p < 0.001.

The effect of partial and complete deletion of tau exon 10 on general behaviour and cognitive function was assessed in IntelliCages, a new non-invasive system for behavioural phenotyping [14,15]. Since the behaviours of E10+/− and wild-type mice (E10+/+) in sensorimotor tests were essentially equal, we chose to only examine two experimental groups, 9–10 months-old E10+/− and E10−/− mice. Moreover we wanted to increase group size and statistical power. First, tendency to explore and habituate to novel environment was examined. During the first 2 hours of the study, the LEDs were turned on in two of the four corners. The E10−/− mice (61.2 ± 4.2%, n = 10) spent more time in the illuminated corners than E10+/− mice (46.6 ± 4.8%, n = 9; t(17) = −2.31, p < 0.05). During the first 24 hours, E10+/− mice (64 ± 17, n = 10) tended to perform more visits to the corners than E10−/− mice (28 ± 10, n = 12; Mann Whitney U = 31, p = 0.06). As previously observed, there was a marked diurnal activity pattern, i.e. more activity during the dark phases compared to light phases. There was a significant time-dependent decrease in the number of corner visits (F(13,260) = 7.0, p < 0.001) indicating that E10+/− and E10−/− habituated to the new environment (Figure 4A). Interestingly, during the habituation, E10+/− mice were significantly more active than E10−/− mice (F(1,20) = 8.87, p < 0.01; Figure 4A). The amount of time spent drinking, measured as lick duration, increased over days for both groups of mice (F(13,260) = 6.74, p < 0.001, data not shown), but the length of lick duration did not differ between genotypes. Despite a habituation effect, three E10−/− mice performed few corner visits and drank little, and therefore had to be excluded. They were replaced by four new E10−/− mice, which were allowed to habituate to the IntelliCages for additional 7 days. The inserted mice were initially very active, with high numbers of corner visits. In the following days, the number of corner visits drastically decreased. Their activity was not significantly different from the rest of the mice during the last three days of the habituation.

### Place learning or associative learning do not depend on the presence of exon 10 in tau

The IntelliCages are equipped with four corners, each with two doors leading to their respective water bottle. In the Place learning protocol, an error was defined as a nosepoke on any of the seven doors where sucrose water (reward) was not available, i.e. the closed side of each corner (=four doors) and the accessible side in the three corners where only water was available (=three doors). Any visit to a door where reward was not provided was defined as an erroneous behaviour. The percentage of errors made by E10−/− mice decreased from the first day of testing to the last day (from 84.2 ± 4.6% to 63.6 ± 8.9%, p = 0.06; chance level is 87.5%). Likewise, the percentage of errors decreased for E10+/− mice from 95.3 ± 2.3% to 79.8 ± 6.0% (p < 0.05). However, spatial learning ability did not depend on genotype (Figure 4B). During reversal learning, when reward was provided behind the diagonally opposite door, E10−/− mice made more errors on first 84.2 ± 5.5% than on the last day of testing 59.7 ± 8.7% (p < 0.05; chance level is 87.5%), but this was not observed for E10+/− mice (82.8 ± 5.7% to 79.7 ± 6.2%, p = 0.72; Figure 4C). Again, reversal learning did not depend on genotype. When mice again could access water behind all eight doors (extinction of Place learning, 12 hours), E10+/− and E10−/− mice preferred the previously rewarded location (t(38) = 2.09, p < 0.05 i.e. above chance level 12.5%; Figure 4D), i.e. they still preferred the location where reward had been provided during the reversal learning. To assess cognitive function, E10+/− and E10−/− mice were also exposed to a passive avoidance test with a memory probe trial [16]. At baseline, before being exposed to an aversive stimulus, neither of the gene-manipulated mice preferred any of the four corners (Figure 4E; left). During training, the mice avoided the corner in which they were exposed to an aversive stimuli (an air-puff), but there was no genotype-dependent difference (training t(18) = 1.07, p = 0.30; probe trial t(18) = 0.35, p = 0.73; chance level is 25%; Figure 4E; middle). In the probe trial, both E10+/− and E10−/− mice avoided the corner where they had previously been exposed to an aversive stimuli (training t(38) = 11.9, p < 0.001; probe trial t(38) = 9.84, p < 0.001; chance level is 25%; Figure 4E; right). These findings were indicative of learning and memory retention abilities in both groups of gene-manipulated mice.

### No genotype-dependent effects on anxiety-like or exploratory behaviours

**Figure 5:**
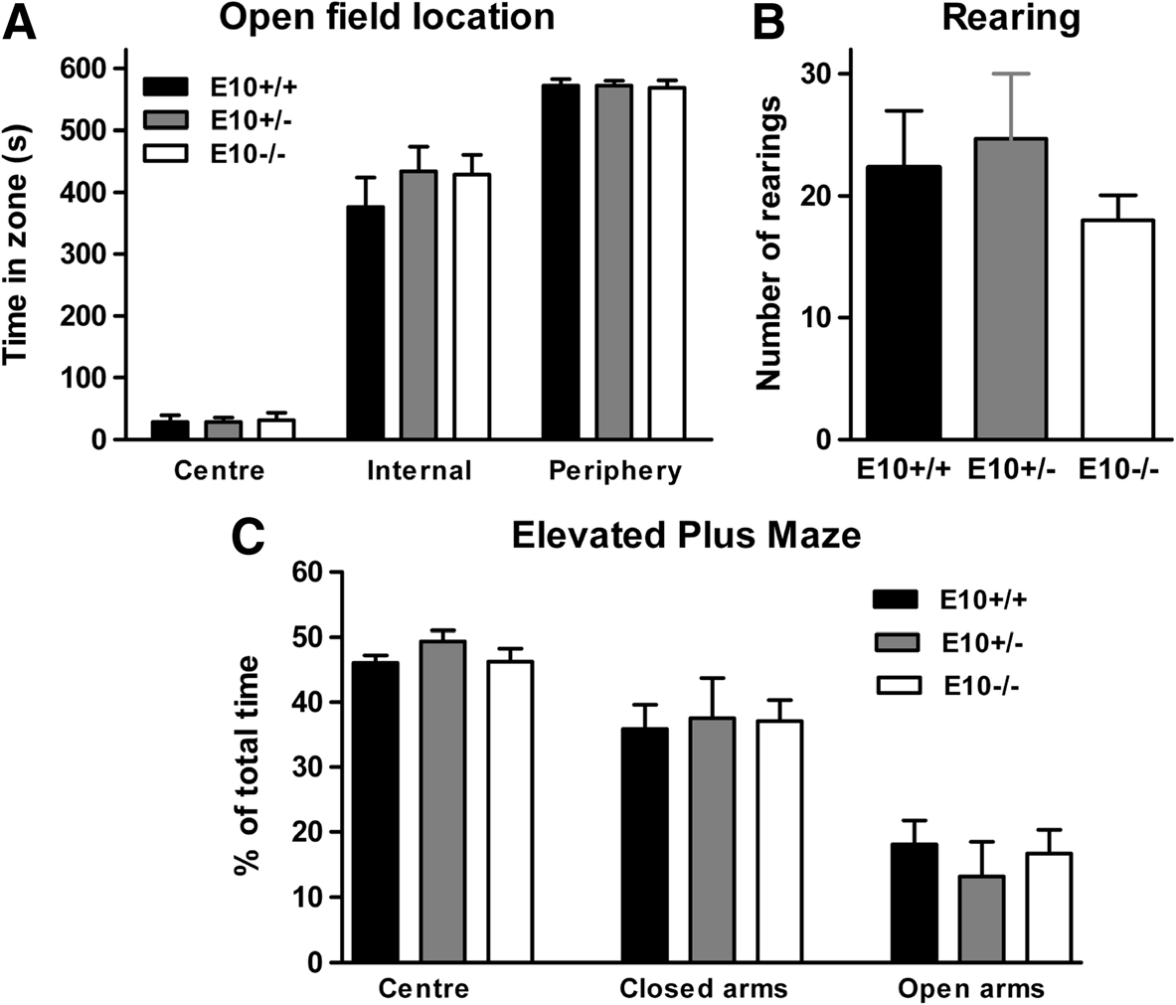
**Exploratory behaviour of wild-type, E10+/− and E10−/− mice. (A)** 12–16 months-old wild-type (E10+/+; n = 9), E10+/− (n = 7) and E10−/− (n = 6) mice explored the different compartments of the open field apparatus to the same extent. **(B)** Rearing activity was similar between the genotypes. **(C)** Behaviour in the elevated plus maze did not depend upon partial or complete ablation of exon 10.

Since, E10−/− mice were less active than E10+/− mice in the intelliCages we wanted to further examine locomotion, emotionality and explorative behaviours with traditional behavioural tests. Behaviour in elevated plus maze is thought to reflect anxiety and risk aversion, while open field measures activity and willingness to explore novel environment. There were no genotype-dependent differences in the open field test. The different mice did not differ in terms of location preference (center F(2) = 0.03, p = 0.97); internal F(2) = 0.04, p = 0.96); periphery F(2) = 0.60, p = 0.56; Figure 5A), distance moved (Additional file 1: Figure S3) or rearing activity (F(2) = 0.49, p = 0.62; Figure 5B). Similarly in the elevated plus maze, gene-manipulated mice were equally unwilling to spend time in the open arms as wild-type (E10+/+) mice (center F(2) = 1.39, p = 0.27); closed arms F(2) = 0.038, p = 0.96); open arms F(2) = 0.36, p = 0.70; Figure 5C).

### Deletion of exon 10 does not result in gross morphological abnormalities or tauopathy

**Figure 6:**
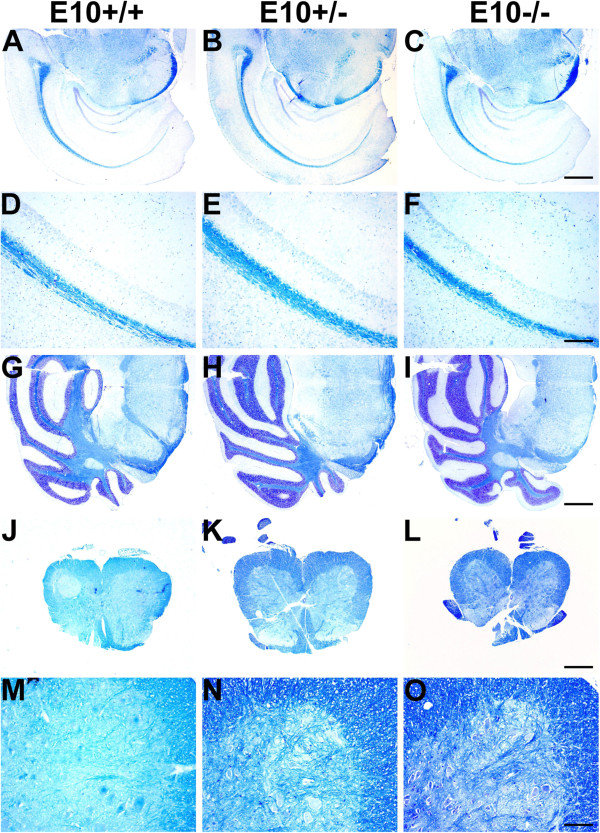
**Gross morphology of wild-type, E10+/− and E10−/− mice.** Coronal sections from 12 months-old mice which were stained with hematoxylin and Luxol fast blue dyes. There were no apparent genotype-dependent differences in **(A-F)** forebrain, **(G-I)** hind brain or **(J-O)** spinal cord. Scale bar measures 500 μm **(A-C, G-I)**, 250 μm **(J-L)**, 125 μm **(D-F)** and 63 μm **(M-O)**.

**Figure 7:**
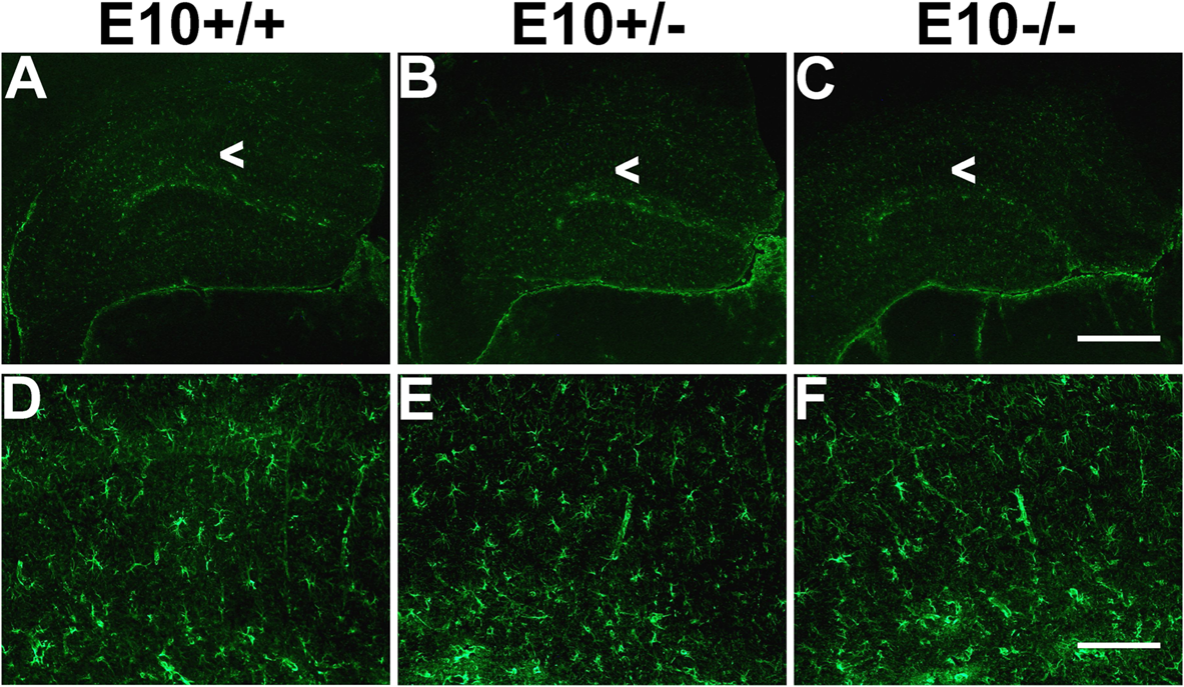
**Astroglial immunostaining in the hippocampus of wild-type, E10+/− and E10−/− mice.** Coronal sections from 12 months-old mice were stained with a GFAP-antibody. Differences in glial staining were not apparent in E10+/− and E10−/− mice when compared to wild-type (E10+/+) mice. Magnification of CA1 region is marked with arrowheads. Scale bars measure **(A-C)** 500 μm or **(D-F)** 125 μm.

Brains of 12 months-old wild-type (E10+/+), E10+/− and E10−/− mice were examined for major structural changes. Sections were stained with hematoxylin and luxol fast blue dyes, but we did not find any genotype-dependent differences in the forebrain, cerebellum or spinal cord (Figure 6). The astroglial marker, glial fibrillary acidic-protein (GFAP) was used as a rather unspecific sensor of abnormalities in brain tissue. However, we did not find any differences between the mice (Figure 7). Similarly, there were no genotype-dependent differences in GFAP-staining in cerebellar tissue (data not shown) and no macroscopic differences of whole brain (Additional file 1: Figure S4). We were unable to find any evidence of tau aggregation in the brains of E10+/− or E10−/− mice (data not shown).

## Discussion

The cytoskeleton helps cells maintain a dynamic cellular morphology in response to the demands of the surrounding environment. Microtubule-binding proteins likely play a major role in cytoskeletal integrity which is highly critical to neuronal functions and viability, since it enables bidirectional axonal transport of vesicles and organelles along extended processes. In the adult human brain, splicing of tau protein gives rise to a 1:1 balanced expression of *3R-* and *4R-tau* mRNA, while in rodents only *4R-tau* mRNA is expressed in adult animals. Interestingly, in mouse brain exon 10 is not utilized for protein synthesis until postnatal stages [11,17], when the genome might be less able to activate compensatory mechanisms and mask phenotypes. This is the first study in which the splicing pattern of *tau*, a microtubule-binding protein, has been altered in the genome with the purpose of studying physiological functions of exon 10 *in vivo*. Gene targeting is typically associated with the creation of knockout mice, often generated by ablating the basal promoter and exon 1. There are a few reports in which the technique has been used to solely eliminate a single exon [18]. In the current study, equal amounts of 3R- and 4R-tau protein were synthesized in 2 months-old E10+/− mice while wild-type littermates only expressed 4R-tau protein. Moreover, immunostaining with a 3R-tau selective antibody confirmed axonal localization of tau in genetically manipulated mice (E10+/−). Altogether, gene targeting technique led us to the successful generation of gene-manipulated mice expressing *3R:4R-tau* (E10+/−) and *3R-tau* (E10−/−) mRNA and protein with expected subcellular localization.

Functional studies revealed an age-dependent decline of sensorimotor skills only in middle-aged E10−/− mice. Adult and middle-aged E10+/− mice had similar motor skills in the rotarod test as E10+/+ mice, consistent with haploinsufficiency seldom giving phenotypes in knockout studies. Interestingly, adult (5–6 months) E10+/− mice were actually stronger than E10+/+ mice, which could be explained by activation of compensatory mechanisms. It has been suggested that other microtubule-associated proteins, MAP1A and MAP1B, are involved in functional compensation. Ablation of *tau* resulted in ~2-fold increased MAP1A levels in young mice (~2 weeks old) [2,4], while levels of MAP1B and other MAPs remained unchanged. Compensatory mechanisms seem more active during embryogenesis and early postnatal development, since MAP1A was unchanged in 12 months-old tau knockout mice [4]. We speculate that functional compensation in adult E10−/− mice was overridden by the effects of aging, resulting in sensorimotor deficits.

Cognitive and emotional functions in the gene-manipulated mice were examined, partly because tau anomalies are related to dementia disorders. We decided to restrict IntelliCage analyses to two groups of mice; E10+/− and E10−/− because we wanted to attain a reasonable statistical power. The decision was also due to availability of IntelliCages and female mice well matched for age. Moreover E10+/− and E10+/+ mice had behaved essentially the same in the sensorimotor tests. These observations were consistent with many other knockout studies, in which haploinsufficiency seldom results in phenotypes. E10−/− mice were less active than E10+/− mice in the IntelliCages, observations which were not reflected in differential anxiety-like behaviours or diminished exploratory behaviour when animals were tested in open field or elevated plus maze. In IntelliCages, mice are housed for days undisturbed in social groups in an environment to which they are allowed to habituate. The procedures of open field and elevated maze testing are markedly different, and this can of course have an impact on the outcome. Both groups habituated well to the new environment and did not show overt cognitive dysfunctions in IntelliCages. We conclude that deletion of exon 10 in *tau* does not result in cognitive or emotional phenotypes, although it remains to be investigated if this occurs with aging.

Taken together phenotypes in mice genetically manipulated around exon 10 in *tau* were limited to sensorimotor defects and partly similar to those found in tau knockout mice [3,19]. Impaired performance might relate to cerebellar dysfunctions, since this brain region to a large extent develops at postnatal stages when splicing of *tau-*mRNA is abnormal in E10+/− and E10−/− mice [20]. We were unable to find gross morphological changes, but there could be subtle differences in neural tree or synaptodendritic connections leading to network dysfunctions. Electrophysiological and high-resolution quantitative morphology would be needed to investigate such changes, studies which we consider to be outside the scope of an initial characterization of the models.

There is evidence to suggest that in tauopathies, the pathological aggregation of tau can be primary pathogenic e.g. in frontotemporal lobe dementia (FTLD-17) or secondary pathogenic e.g. to Aβ-aggregation in AD [21]. However, it remains unclear why human Aβ accumulation by itself does not generate neurofibrillary tangles in AβPP transgenic mice. Perhaps structural differences make murine tau unable to form fibrils but *in vitro* experiments contradict this theory [22]. Murine tau can take part in tau aggregation *in vivo* but so far seems unable to initiate tau pathology by itself [23-25]. An unknown factor or a trigger, such as misfolded or aggregated human tau or perhaps Aβ seems to be critical. The differences in splicing of tau in mouse and human brain, i.e. the 1:1 balanced *3R-/4R-tau* mRNA splicing pattern, could directly impact on the stability of the microtubule structure and on aggregation of tau. It remains to be investigated whether the microtubule system of E10+/− and E10−/− mice is more vulnerable to a trigger of tau-misfolding e.g. an aggregate of human Aβ or human tau than wild-type mice harbouring intact murine *tau* (E10+/+).

## Conclusions

This is the first functional study of exon 10 in *tau*, which is linked to neurodegenerative disease. By deleting exon 10 it was possible to alter alternative splicing of *tau* such that 3R-tau protein was synthesized instead of 4R-tau protein. The anatomic and subcellular localization of tau protein synthesis was maintained in the gene-manipulated animals. Middle-aged mice lacking exon 10 in tau (E10−/−), expressed only 3R-tau protein and displayed mild sensorimotor deficits with reduced grip strength and motor coordination. Haploinsufficient mice (E10+/−), expressing both 3R- and 4R-tau protein, behaved nearly always equal to wild-type mice (E10+/+) which expressed only 4R-tau protein. There were no genotype-dependent effects on cognition, emotionality or on gross brain structure and tissue morphology. Thus, sensorimotor functions depend on *tau* exon 10, while perhaps additional functions are masked by related microtubule-binding proteins.

## Methods

### Ethical note

Appropriate experimental procedures were approved by the committee of ethical conduct in research on animal at the Uppsala University (ethical permits C17/7 (2007-03-02 – 2010-03-02), C374/9 (2010-01-29 – 2013-01-29) and C278/12 (2012-12-20 – present) and performed according to guidelines of ethical conduct on science in compliance with local animal care.

### Gene targeting

A 2.2-kbp genomic fragment in intron 9 of the murine *tau* gene was amplified with genomic DNA from R1 embryonic stem (ES) cells as template using high-fidelity Expand PCR system (Roche, Basel, Switzerland). Primers started at 15718139 and 15720351 respectively in NT_165773.2. Likewise a 3.6-kbp fragment stretching from intron 10 into intron 11 was amplified with primers starting at 15720889 and 15724540 in NT_165773.2. Primers were designed as to avoid ~200 bp intron sequences located in introns 9 and 10, immediately adjacent to exon 10. Both genomic fragments were inserted into the pBluescript II KS-vector where they flanked a 2 kbp fragment containing a neomycin gene surrounded by loxP-sites that had been excised from the pKK7-vector. The targeting construct was checked by multiple restriction enzyme digests and fully sequenced. Only a few mismatches were found as compared to the NCBI database. None of those were located in or adjacent to protein coding sequences. The targeting construct was introduced into R1 ES cells by electroporation and homologous recombination was verified in 7 out of 375 individual clones by screening with two long-range PCR reactions. In both reactions, one primer annealed to sequences outside the targeted allele (starting at 15717864 and 15724785 respectively) while the other primer annealed to sequences within the neomycin (neo) gene. Male chimeras were obtained by microinjection of ES cell clones into blastocysts and subsequent transplantation of these blastocysts to CD1 pseudopregnant mice. The blastocysts were generated by mating with C57BL/6NCrl females and B6D2F1/Crl males, Male chimeras were bred with female C57Bl/6JBomTac mice (Taconic, Hudson, NY, USA) as to generate female offspring, which were then bred with male transgenic mice expressing phosphoglycerate kinase (pgk)-CRE recombinase (BALB/c × C57B1/B6)F1 [26]. The loxP-flanked neo-cassette was thereby deleted from the targeted allele in some of the offspring.

### PCR analyses of genomic DNA and mRNA

Genomic DNA from ear-biopsies was prepared as previously described [27]. PCR amplification was done with primers annealing at 15720175 and 15721073 in NT_165773.2 as to frame exon 10 in the murine *tau* gene and to generate ~900 or ~460 bp bands depending upon the inclusion or exclusion of exon 10. Alternatively the targeted allele was identified by using with one primer located in the ~100 bp sequence adjacent to the remaining loxP-site, and therefore unique to the targeted allele, and the other primer in nearby intron sequences of the murine *tau* gene. Total RNA was purified with RNA STAT-60 (Tel-Test, Friendwood, TX, USA), which is based on the guanidine thiocyanate method. Universal Riboclone cDNA synthesis system (Promega, Madison, WI, USA) and an antisense primer located at 1281–1262 in NM_010838.3 were used for first strand synthesis. The nascent cDNA was then PCR amplified with primers which annealed at 671–690 (in exon 9) and 975–954 (in exon 11) in NM_010838.3 as to simultaneously assay *3R-tau* and *4R-tau* mRNA expression. Alternatively cDNA was amplified with primers located at 671–690 (in exon 9) and 893–876 (in exon 10) whereby only *4R-tau* mRNA was detected. The PCR products were separated by electrophoresis on ethidium bromide-stained agarose gels, and examined and photographed under UV-light.

### Histology and western blot

Mice were anesthetized with 0.4 ml Avertin (25 mg/ml) and intracardially perfused with 0.9% (w/v) saline solution. Brains were dissected and either directly frozen on dry ice for western blot analyses, or instantly frozen in 2-methylbutane at −30°C with dry ice and used for immunohistochemistry. All tissues were stored at −80°C until use. Sections, 14 μm, were cut in a cryostat, fixed in 4% formalin for 5 min, permeabilized in 0.4% Triton X-100 for 5 min and treated with 0.3% H_2_O_2_ for 15 min to eliminate endogenous peroxidase activity. Tissues were then incubated with DAKO-block for 1 h to block unspecific signal and then with primary antibodies 3RD (x40) and Tau-1 (×200) (Millipore, Billerica, MA, USA) over night at +4°C. Sections were then washed and treated with secondary antibodies, ABC-reagents and developed with NOVA RED as described [28]. For Cresyl violet and Luxol fast-blue staining, mice were intracardially perfused with 4% (w/v) formaldehyde solution and neuronal tissues were dissected and immersed in this solution for 3 days, dehydrated in etOH (40% 2×4 h; 70% o.n.; 80% 2×1.5 h; 95% 2×1.5 h; 100% 2×1 h; Xylen 2×30 min), immersed in paraffin o.n at 62°C, embedded and sectioned at 5 μm. Brains used for western blot were extracted in 10 mM Tris, 0.8 M NaCl, 1 mM EDTA, 10% (w/v) sucrose supplemented with Complete® (1:50) protease inhibitor cocktail (Roche, Basel, Switzerland) and centrifuged at 26 000 × *g* for 20 min at +4°C. The pellet was extracted and centrifuged again at 26 000 × *g* for 20 min at +4°C. The supernatants were pooled and supplemented with 1% (w/v) sarkosyl and incubated by rotary shaking at 200 RPM for 1 h at RT. The mixture was then centrifuged at 100 000 × *g* for 1 h at +4°C and the sarkosyl-soluble supernatants were run on 8% Tris-Glycine gels (InVitrogen, Carlsbad, CA, USA) and analyzed with western blot as previously described [28]. A human recombinant tau protein ladder was used as size markers (rPeptide, Bogart, GA, USA). Nitrocellulose filters were incubated with primary antibodies 3RD (x20 000) and Tau-1 (x50 000) in 5% (w/v) non-fat dry milk in 1×TTBS for 1 h at RT.

### Rotarod

Motor coordination of wild-type (E10+/+), E10+/− and E10−/− mice was assessed at 5–7 months and 13–17 months of age. The ROTA-ROD apparatus (dimensions 362 × 240 × 400 mm) (model LE8200, Panlab, S.L.U., Spain) consisted of a vertical plastic (methacrylate) rotating rod (dimensions 50 × 30 mm) with a ribbed surface flanked by two large discs (arnite). Each mouse was given three trials with 10 min inter-trial interval for three consecutive days. Trials started at a minimum speed of 4 rpm followed by 5 min acceleration time (maximum speed 40 rpm) with increase of speed with 1 rpm every 8^th^ second. The latency to fall and the rotation speed when the mice fell was recorded. The apparatus was cleaned with 70% ethanol between trials.

### Grip strength

Muscular strength of wild-type (E10+/+), E10+/− and E10−/− mice was evaluated at 5–7 months and 13–17 months of age. The grip strength meter (model GS3, BIOSEB, Chaville, France) consisted of a stainless steel grid (dimensions 100 × 80 mm) with a sensor capacity ranging 0–20 Newton (N). Each mouse was held by the tail, placed on the grid with all four paws and gently pulled horizontally by the tail along a straight line until the mice released the grid and the maximum forced was recorded. Each mouse was given three trials with 30 seconds inter-trial interval for four consecutive days. The stainless grid was cleaned with 70% ethanol between trials.

### IntelliCage

Behaviours of female E10+/− and E10−/− mice housed in social groups were tested at 9–11 months of age. The protocols used have been previously described in detail [14]. Each IntelliCage was equipped with four learning corners accessible through a ring antenna which led to two mechanical doors controlling access to two water bottles. Light-emitting diodes (LEDs; colour code 1638) were located above each door inside the learning corners. Three events were recorded in the learning corners; visit, nosepoke and lick. A visit was recorded when the ring antenna detected the microchip and the heat sensor simultaneously sensed a temperature shift. A nosepoke was recorded when the infrared beam in front of the mechanical door was interrupted. A lick was recorded when a mouse touched the nipple of the water bottle such that the “lick sensor” exceeded a threshold level. IntelliCage software (ver. 2.4.2) and IntelliCage corners (ver. 2.0) were used (http://www.newbehavior.com). The animals were allowed to habituate to the testing room for a week, and were then under isoflurane anaesthesia subcutaneously injected with transponders in the interscapular area. At least 24 h after transponder implantation, the mice were placed into the IntelliCages at the beginning of the dark phase. The duration of IntelliCage housing was 6 weeks, during which the dark phase was scheduled from 20:00 until 08:00 and light phase 08:00–20:00. The modules, which are listed in Table 1, have previously been extensively reported [14], and here only deviations from those protocols are described:

**Table 1:** Behavioural analyses in the IntelliCage

**Name of the module**	**Duration (days)**
1. Free exploration (FE)	1
2. Habituation	14
3. Place learning (PL)	4
4. Reversal place learning (RevPL)	4
5. Extinction of place preference	2
6. Passive avoidance	3

A) Place learning (PL): In a designated corner for each mouse, a nosepoke opened the left door once during a visit. Thereby the animals were given access to 300 mM sucrose (rewarded corner) during two drinking sessions per night (23:00–24:00 and 04:00–05:00). The right door in the same corner remained closed. In the other three corners, a nosepoke opened the right door which gave access to water (unrewarded corner) once during the two drinking sessions. The left door remained closed during the rest of time. The corner in which reward was given was randomized and individual designated to each mouse.

(B) Reversal learning during drinking sessions (revPL): The same protocol as PL except that the respective corner giving access to sucrose was the diagonal opposite.

### Elevated plus maze

Anxiety-like behaviours of 12–16 months-old wild-type (E10+/+), E10+/− and E10−/− mice were assessed in a maze made of grey coloured PVC walls (model LE842, Panlab S.L.U., Barcelona, Spain). It consisted of two open arms and two closed arms (length 29.5 cm and width 6 cm) arranged as a cross. The center was 6 × 6 cm and the closed arms were protected by a 15 cm wall made of grey PVC. Individual mice were placed at the center of the maze facing the open arms for a single 5 min trial. Their behaviours were recorded on video and analysed with Smart Junior program V1.0. The maze was cleaned with 70% ethanol between trials.

### Open-field test

A transparent cage (width 37 cm, length 48 cm and height 20 cm) was used to assess locomotor activity, exploratory and anxiety-like behaviours of 12–16 months-old wild-type (E10+/+), E10+/− and E10−/− mice. Individual mice were placed at the center of the cage for 10 min and their behaviour was recorded with a video camera. The testing cage was divided in three zones: periphery, internal and center comprising 43.6%, 48.6% and 7.8% of the total area respectively. Off-line analysis of the records was performed with Smart Junior program V1.0. The area was cleaned with 70% ethanol between trials.

### Statistical analyses

GraphPad (San Diego, CA, USA) and Statistica (Tulsa, OK, USA) were used. Data which had been verified not to deviate from a normal distribution were analyzed with unpaired *t-*test, one-way or two-way ANOVA, or otherwise with a non-parametric test e.g. Mann–Whitney U-test. Outliners were identified with Grubbs’ test, and post-hoc test of one-way and two-way ANOVA were analyzed with Fisher LSD. Results presented are mean ± s.e.m. unless otherwise stated.

## Abbreviations

AβPP: Amyloid-β precursor protein; Aβ: Amyloid-β; CA1: Cornu ammonis 1; CRE: Causes recombination; E10: Exon 10; ES: Embryonic stem; FE: Free exploration; FTLD-17: Frontotemporal lobe dementia (FTLD-17); GFAP: Glial fibrillary acidic-protein; LEDs: Light emitting diodes; MAPs: Microtubule-associated proteins; NCBI: National center for biotechnology information; RT-PCR: Reverse transcriptase polymerase chain reaction; 3R: 3 repeat; pgk: Phosphoglycerate kinase; P1: Postnatal day 1; PL: Place learning; PVC: Polyvinyl chloride.

## Competing interests

In this study there are no actual or potential conflicts of interests.

## Authors’ contributions

LN conceived the study, designed and generated the gene targeting vector and screened embryonic stem cell clones. Uppsala University Transgenic Facility cultured embryonic stem cells and performed blastocyst injection and implantation. AG was responsible for animal care and breeding. She planned and carried out behavioural studies and associated data analyses; LN and AG carried out immunohistochemical and biochemical experiments, and together designed experiments and wrote ethical applications. AG did statistical analyses under the supervision of LN. LN, AG and LL wrote the manuscript. All authors read and approved the final manuscript.

## Supplementary Material

Additional file 1: Figure S1 A graphical illustration of the genomic structure around exon 10 on both alleles in the murine *tau* gene locus, *tau* mRNA expression and tau protein synthesis in wild-type (E10+/+, upper), E10+/- (middle) and E10-/- mice (lower). A part of the *tau* locus with exons 9, 11 (yellow), 10 (orange, dotted lines) and genetic deletion (►) is shown. The four microtubule domains (R1-R4) in tau protein are encoded by exons 9-12 in *tau*. Alternative splicing of *tau* in adult wild-type mouse brain results in 4R-tau protein being synthesized by both alleles (upper). Ablation of exon 10 on one of the two alleles in *tau* (E10+/- mice) should theoretically lead to a 1:1 balanced ratio of 3R- and 4R-tau protein synthesis (middle). This mixture of 3R-/4R-tau protein synthesis is found in adult human brain. In contrast, lack of exon 10 on both alleles in *tau* (E10-/-) should results in 3R-tau protein synthesis by both alleles (lower). **Figure S2.** Sensorimotor functions of wild-type (E10+/+), E10+/- and E10-/- mice. 13-17 months-old mice devoid of *tau* exon 10 (E10-/-) were also impaired in rotarod when maximum speed was recorded compared to E10+/+ (p < 0.05) and E10+/- mice (p < 0.01) (E10+/+, n = 18; E10+/-, n = 16; E10-/-; n = 15). *p < 0.05 and **p < 0.01. **Figure S3.** Exploratory behaviours of wild-type E10(+/+), E10+/- and E10-/- mice. At 12-16 months of age, E10+/- and E10-/- mice travelled a similar distance in the open field apparatus as wild-type mice. **Figure S4.** No macroscopic differences between brains of wild-type (E10+/+) and E10+/- and E10-/- mice. Click here for file

## References

[B1] Dehmelt L Halpain S The MAP2/Tau family of microtubule-associated proteins Genome Biol 2005 6 1 204 15642108 10.1186/gb-2004-6-1-204 PMC549057

[B2] Harada A Oguchi K Okabe S Kuno J Terada S Ohshima T Sato-Yoshitake R Takei Y Noda T Hirokawa N Altered microtubule organization in small-calibre axons of mice lacking tau protein Nature 1994 369 6480 488 491 10.1038/369488a0 8202139

[B3] Ikegami S Harada A Hirokawa N Muscle weakness, hyperactivity, and impairment in fear conditioning in tau-deficient mice Neurosci Lett 2000 279 3 129 132 10.1016/S0304-3940(99)00964-7 10688046

[B4] Dawson HN Ferreira A Eyster MV Ghoshal N Binder LI Vitek MP Inhibition of neuronal maturation in primary hippocampal neurons from tau deficient mice J Cell Sci 2001 114 Pt 6 1179 1187 11228161 10.1242/jcs.114.6.1179

[B5] Takei Y Teng J Harada A Hirokawa N Defects in axonal elongation and neuronal migration in mice with disrupted tau and map1b genes J Cell Biol 2000 150 5 989 1000 10.1083/jcb.150.5.989 10973990 PMC2175245

[B6] Andreadis A Tau gene alternative splicing: expression patterns, regulation and modulation of function in normal brain and neurodegenerative diseases Biochim Biophys Acta 2005 1739 2–3 91 103 15615629 10.1016/j.bbadis.2004.08.010

[B7] Janke C Beck M Stahl T Holzer M Brauer K Bigl V Arendt T Phylogenetic diversity of the expression of the microtubule-associated protein tau: implications for neurodegenerative disorders Brain Res Mol Brain Res 1999 68 1–2 119 128 10320789 10.1016/s0169-328x(99)00079-0

[B8] Hutton M Lendon CL Rizzu P Baker M Froelich S Houlden H Pickering-Brown S Chakraverty S Isaacs A Grover A Association of missense and 5′-splice-site mutations in tau with the inherited dementia FTDP-17 Nature 1998 393 6686 702 705 10.1038/31508 9641683

[B9] Hong M Zhukareva V Vogelsberg-Ragaglia V Wszolek Z Reed L Miller BI Geschwind DH Bird TD McKeel D Goate A Mutation-specific functional impairments in distinct tau isoforms of hereditary FTDP-17 Science 1998 282 5395 1914 1917 9836646 10.1126/science.282.5395.1914

[B10] Kosik KS Orecchio LD Bakalis S Neve RL Developmentally regulated expression of specific tau sequences Neuron 1989 2 4 1389 1397 10.1016/0896-6273(89)90077-9 2560640

[B11] Takuma H Arawaka S Mori H Isoforms changes of tau protein during development in various species Brain Res Dev Brain Res 2003 142 2 121 127 10.1016/S0165-3806(03)00056-7 12711363

[B12] Feinstein SC Wilson L Inability of tau to properly regulate neuronal microtubule dynamics: a loss-of-function mechanism by which tau might mediate neuronal cell death Biochim Biophys Acta 2005 1739 2–3 268 279 15615645 10.1016/j.bbadis.2004.07.002

[B13] Binder LI Frankfurter A Rebhun LI The distribution of tau in the mammalian central nervous system J Cell Biol 1985 101 4 1371 1378 10.1083/jcb.101.4.1371 3930508 PMC2113928

[B14] Codita A Gumucio A Lannfelt L Gellerfors P Winblad B Mohammed AH Nilsson LN Impaired behavior of female tg-ArcSwe APP mice in the IntelliCage: a longitudinal study Behav Brain Res 2010 215 1 83 94 10.1016/j.bbr.2010.06.034 20615433

[B15] Galsworthy MJ Amrein I Kuptsov PA Poletaeva II Zinn P Rau A Vyssotski A Lipp HP A comparison of wild-caught wood mice and bank voles in the IntelliCage: assessing exploration, daily activity patterns and place learning paradigms Behav Brain Res 2005 157 2 211 217 10.1016/j.bbr.2004.06.021 15639172

[B16] Voikar V Colacicco G Gruber O Vannoni E Lipp HP Wolfer DP Conditioned response suppression in the IntelliCage: assessment of mouse strain differences and effects of hippocampal and striatal lesions on acquisition and retention of memory Behav Brain Res 2010 213 2 304 312 10.1016/j.bbr.2010.05.019 20493907

[B17] McMillan P Korvatska E Poorkaj P Evstafjeva Z Robinson L Greenup L Leverenz J Schellenberg GD D’Souza I Tau isoform regulation is region- and cell-specific in mouse brain J Comp Neurol 2008 511 6 788 803 10.1002/cne.21867 18925637 PMC2845852

[B18] Gimond C Baudoin C van der Neut R Kramer D Calafat J Sonnenberg A Cre-loxP-mediated inactivation of the alpha6A integrin splice variant in vivo: evidence for a specific functional role of alpha6A in lymphocyte migration but not in heart development J Cell Biol 1998 143 1 253 266 10.1083/jcb.143.1.253 9763436 PMC2132821

[B19] Roberson ED Scearce-Levie K Palop JJ Yan F Cheng IH Wu T Gerstein H Yu GQ Mucke L Reducing endogenous tau ameliorates amyloid beta-induced deficits in an Alzheimer’s disease mouse model Science 2007 316 5825 750 754 10.1126/science.1141736 17478722

[B20] Kolb B Tees RC The Cerebral cortex of the rat 1990 Cambridge, Mass: MIT Press

[B21] Ballatore C Lee VM Trojanowski JQ Tau-mediated neurodegeneration in Alzheimer’s disease and related disorders Nat Rev Neurosci 2007 8 9 663 672 17684513 10.1038/nrn2194

[B22] Kampers T Pangalos M Geerts H Wiech H Mandelkow E Assembly of paired helical filaments from mouse tau: implications for the neurofibrillary pathology in transgenic mouse models for Alzheimer’s disease FEBS letters 1999 451 1 39 44 10.1016/S0014-5793(99)00522-0 10356980

[B23] Mocanu MM Nissen A Eckermann K Khlistunova I Biernat J Drexler D Petrova O Schonig K Bujard H Mandelkow E The potential for beta-structure in the repeat domain of tau protein determines aggregation, synaptic decay, neuronal loss, and coassembly with endogenous Tau in inducible mouse models of tauopathy J Neurosci 2008 28 3 737 748 10.1523/JNEUROSCI.2824-07.2008 18199773 PMC6670355

[B24] de Calignon A Polydoro M Suarez-Calvet M William C Adamowicz DH Kopeikina KJ Pitstick R Sahara N Ashe KH Carlson GA Propagation of tau pathology in a model of early Alzheimer’s disease Neuron 2012 73 4 685 697 10.1016/j.neuron.2011.11.033 22365544 PMC3292759

[B25] Liu L Drouet V Wu JW Witter MP Small SA Clelland C Duff K Trans-synaptic spread of tau pathology in vivo PloS one 2012 7 2 e31302 10.1371/journal.pone.0031302 22312444 PMC3270029

[B26] Lallemand Y Luria V Haffner-Krausz R Lonai P Maternally expressed PGK-Cre transgene as a tool for early and uniform activation of the Cre site-specific recombinase Transgenic Res 1998 7 2 105 112 10.1023/A:1008868325009 9608738

[B27] Conner DA Mouse colony management Curr Protoc Mol Biol 2002 57 23.8.1 23.8.11 10.1002/0471142727.mb2308s57 18265310

[B28] Lord A Kalimo H Eckman C Zhang XQ Lannfelt L Nilsson LN The Arctic Alzheimer mutation facilitates early intraneuronal Abeta aggregation and senile plaque formation in transgenic mice Neurobiol Aging 2006 27 1 67 77 10.1016/j.neurobiolaging.2004.12.007 16298242

