# Supplementary material for: Lack of exon 10 in the murine tau gene results in mild sensorimotor defects with aging
Source: BMC Neurosci. 2013 Nov 22;14:148. doi: 10.1186/1471-2202-14-148 (PMC4222861; doi:10.1186/1471-2202-14-148)
Supplement: Additional file 1: Figure S1 — A graphical illustration of the genomic structure around exon 10 on both alleles in the murine tau gene locus, tau mRNA expression and tau protein synthesis in wild-type (E10+/+, upper), E10+/- (middle) and E10-/- mice (lower). A part of the tau locus with exons 9, 11 (yellow), 10 (orange, dotted lines) and genetic deletion (►) is shown. The four microtubule domains (R1-R4) in tau protein are encoded by exons 9-12 in tau. Alternative splicing of tau in adult wild-type mouse brain results in 4R-tau protein being synthesized by both alleles (upper). Ablation of exon 10 on one of the two alleles in tau (E10+/- mice) should theoretically lead to a 1:1 balanced ratio of 3R- and 4R-tau protein synthesis (middle). This mixture of 3R-/4R-tau protein synthesis is found in adult human brain. In contrast, lack of exon 10 on both alleles in tau (E10-/-) should results in 3R-tau protein synthesis by both alleles (lower). Figure S2. Sensorimotor functions of wild-type (E10+/+), E10+/- and E10-/- mice. 13-17 months-old mice devoid of tau exon 10 (E10-/-) were also impaired in rotarod when maximum speed was recorded compared to E10+/+ (p < 0.05) and E10+/- mice (p < 0.01) (E10+/+, n = 18; E10+/-, n = 16; E10-/-; n = 15). *p < 0.05 and **p < 0.01. Figure S3. Exploratory behaviours of wild-type E10(+/+), E10+/- and E10-/- mice. At 12-16 months of age, E10+/- and E10-/- mice travelled a similar distance in the open field apparatus as wild-type mice. Figure S4. No macroscopic differences between brains of wild-type (E10+/+) and E10+/- and E10-/- mice. [file 1471-2202-14-148-S1.pdf]

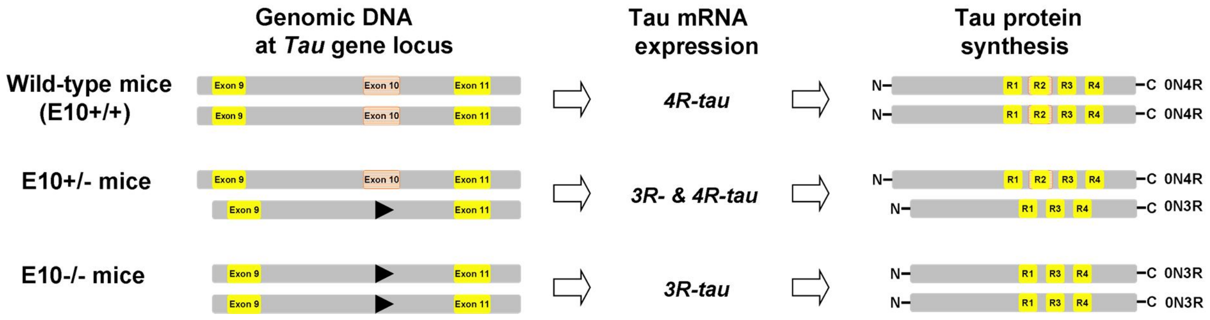

Additional file 1: Figure S1. A graphical illustration of the genomic structure around exon 10 on both alleles in the murine *tau* gene locus, *tau* mRNA expression and tau protein synthesis in wild-type (E10+/+, upper), E10+/- (middle) and E10-/- mice (lower). A part of the *tau* locus with exons 9, 11 (yellow), 10 (orange, dotted lines) and genetic deletion (▶) is shown. The four microtubule domains (R1-R4) in tau protein are encoded by exons 9-12 in *tau*. Alternative splicing of *tau* in adult wild-type mouse brain results in 4R-tau protein being synthesized by both alleles (upper). Ablation of exon 10 on one of the two alleles in *tau* (E10+/- mice) should theoretically lead to a 1:1 balanced ratio of 3R- and 4R-tau protein synthesis (middle). This mixture of 3R-/4R-tau protein synthesis is found in adult human brain. In contrast, lack of exon 10 on both alleles in *tau* (E10-/-) should results in 3R-tau protein synthesis by both alleles (lower).

You created this PDF from an application that is not licensed to print to novaPDF printer (http://www.novapdf.com)

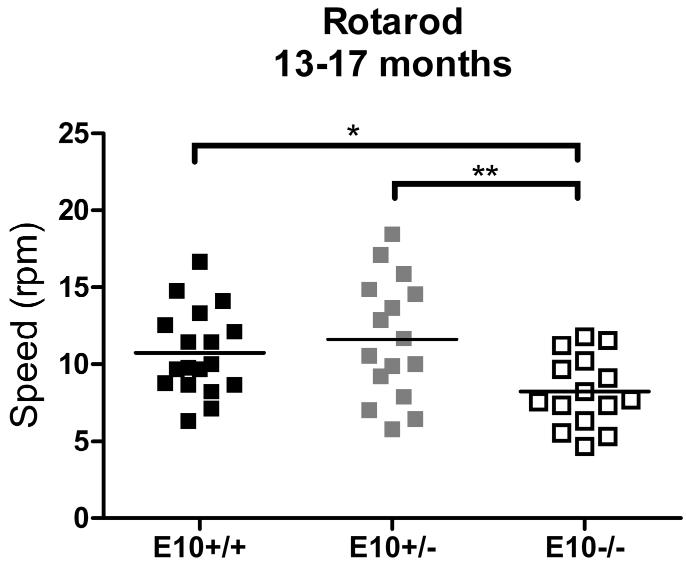

Additional file 1: Figure 2. Sensorimotor functions of wild-type (E10+/+), E10+/- and E10-/- mice. 13-17 months-old mice devoid of *tau* exon 10 (E10-/-) were also impaired in rotarod when maximum speed was recorded compared to E10+/+ ($p<0.05$) and E10+/- mice ($p<0.01$) (E10+/+, n=18; E10+/-, n=16; E10-/-; n=15). *$p<0.05$ and **$p<0.01$

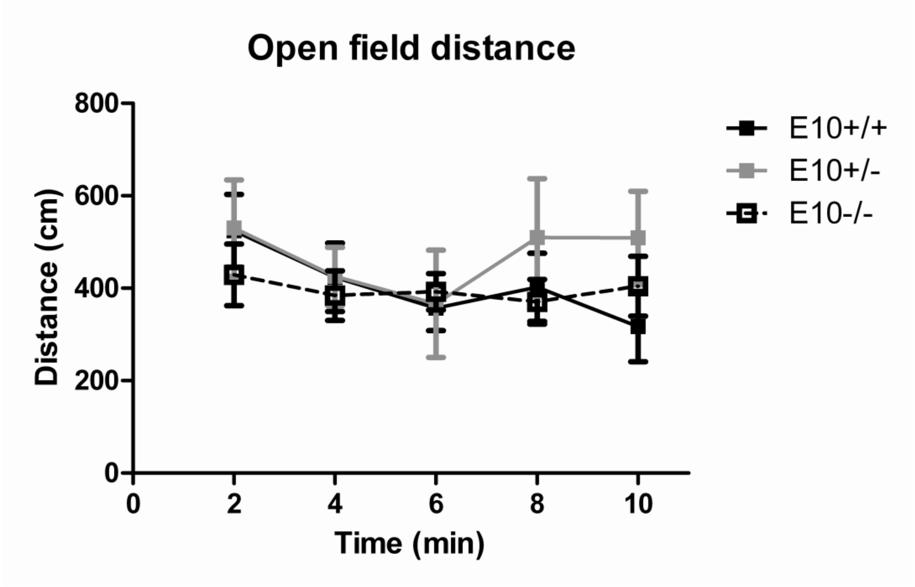

Additional file 1: Figure 3. Exploratory behaviours of wild-type E10(+/+), E10+/- and E10-/- mice. At 12-16 months of age, E10+/- and E10-/- mice travelled a similar distance in the open field apparatus as wild-type mice.

You created this PDF from an application that is not licensed to print to novaPDF printer (http://www.novapdf.com)

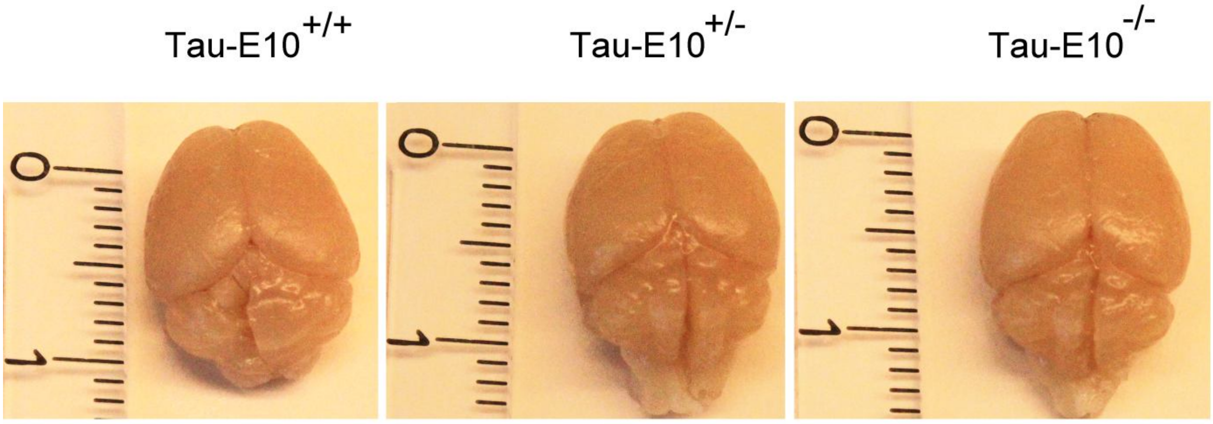

Additional file 1: Figure 4. No macroscopic differences between brains of wild-type (E10+/+) and E10+/- and E10-/- mice.

You created this PDF from an application that is not licensed to print to novaPDF printer (http://www.novapdf.com)
